# Multiphoton Microscopy and Mass Spectrometry for Revealing Metabolic Heterogeneity of Hepatocytes *in vivo*

**DOI:** 10.17691/stm2021.13.2.02

**Published:** 2021-04-30

**Authors:** S.A. Rodimova, D.S. Kuznetsova, N.V. Bobrov, A.A. Gulin, A.A. Vasin, M.V. Gubina, V.I. Scheslavsky, V.V. Elagin, M.M. Karabut, V.E. Zagainov, E.V. Zagaynova

**Affiliations:** Junior Researcher, Laboratory of Regenerative Medicine, Research Institute of Experimental Oncology and Biomedical Technologies, Privolzhsky Research Medical University, 10/1 Minin and Pozharsky Square, Nizhny Novgorod, 603005, Russia; PhD Student, Institute of Biology and Biomedicine, National Research Lobachevsky State University of Nizhni Novgorod, 23 Prospekt Gagarina, Nizhny Novgorod, 603950, Russia; Researcher, Laboratory of Regenerative Medicine, Research Institute of Experimental Oncology and Biomedical Technologies, Privolzhsky Research Medical University, 10/1 Minin and Pozharsky Square, Nizhny Novgorod, 603005, Russia; Assistant, Department of Theoretical Surgery and Transplantology, Privolzhsky Research Medical University, 10/1 Minin and Pozharsky Square, Nizhny Novgorod, 603005, Russia; Surgeon, Oncology Department, Volga District Medical Centre of Federal Medical Biological Agency of Russia, 14 Ilyinskaya St., Nizhny Novgorod, 603109, Russia; Senior Researcher, Acting Head of the Laboratory of Biophotonics, N.N. Semenov Federal Research Center for Chemical Physics, Russian Academy of Sciences, 4 Kosygina St., Moscow, 119991, Russia; Researcher, Faculty of Chemistry, Lomonosov Moscow State University, 1 Leninskiye Gory, Moscow, 119991, Russia; Research Engineer, Laboratory of Nanophotonics, N.N. Semenov Federal Research Center for Chemical Physics, Russian Academy of Sciences, 4 Kosygina St., Moscow, 119991, Russia; Student, Faculty of Chemistry, Lomonosov Moscow State University, 1 Leninskiye Gory, Moscow, 119991, Russia; Research Engineer, Laboratory of Nanophotonics, N.N. Semenov Federal Research Center for Chemical Physics, Russian Academy of Sciences, 4 Kosygina St., Moscow, 119991, Russia; Student, Phystech School of Electronics, Photonics and Molecular Physics, Moscow Institute of Physics and Technology (National Research University), 9 Institutskiy per., Dolgoprudny, Moscow Region, 141701, Russia; Senior Researcher, Becker & Hickl, GmbH, Nunsdorfer Ring 7–9, Berlin, 12277, Germany; Head of the Laboratory of High-Resolution Microscopy, Research Institute of Experimental Oncology and Biomedical Technologies, Privolzhsky Research Medical University, 10/1 Minin and Pozharsky Square, Nizhny Novgorod, 603005, Russia; Researcher, Laboratory of High-Resolution Microscopy, Research Institute of Experimental Oncology and Biomedical Technologies, Privolzhsky Research Medical University, 10/1 Minin and Pozharsky Square, Nizhny Novgorod, 603005, Russia; Researcher, Laboratory of Genomics and Adaptive Antitumor Immunity, Privolzhsky Research Medical University, 10/1 Minin and Pozharsky Square, Nizhny Novgorod, 603005, Russia; Head of the Department of Theoretical Surgery and Transplantology, Privolzhsky Research Medical University, 10/1 Minin and Pozharsky Square, Nizhny Novgorod, 603005, Russia; Chief Specialist in Surgery, Volga District Medical Centre of Federal Medical Biological Agency of Russia, 14 Ilyinskaya St., Nizhny Novgorod, 603109, Russia; Rector, National Research Lobachevsky State University of Nizhni Novgorod, 23 Prospekt Gagarina, Nizhny Novgorod, 603950, Russia; Senior Researcher, Research Institute of Experimental Oncology and Biomedical Technologies, Privolzhsky Research Medical University, 10/1 Minin and Pozharsky Square, Nizhny Novgorod, 603005, Russia

**Keywords:** heterogeneity of hepatocytes, metabolic status of hepatocytes, multiphoton microscopy, FLIM, ToF-SIMS

## Abstract

**The aim of the investigation** was to study the possibility of revealing the heterogeneity of normal liver hepatocytes in terms of metabolic status using the modern methods of multiphoton microscopy and mass spectrometry.

**Materials and Methods:**

Heterogeneity of hepatocytes in terms of total metabolic activity was assessed using multiphoton microscopy based on the autofluorescence intensity of intracellular cofactors NAD(P)H and FAD. Hepatocyte heterogeneity in terms of intensity of intracellular metabolic processes was determined using the fluorescence lifetime imaging (FLIM) method based on the data about fluorescence lifetime contributions of various forms of NAD(P)H. The method of time-of-flight secondary ion mass spectrometry (TоF-SIMS) was used to study the lipid and amino acid composition of hepatocytes.

**Results:**

It has been revealed using multiphoton microscopy that hepatocytes are heterogeneous in terms of general metabolic activity. Using FLIM, it was established that the heterogeneity degree was high in terms of intensity of oxidative phosphorylation, glycolysis, and synthetic processes (lipogenesis, nucleic acid synthesis, and the pentose phosphate pathway). The TоF-SIMS method revealed the presence of hepatocyte heterogeneity in terms of amino acid and lipid composition, which points to various intensities of synthetic processes in individual hepatocytes. Moreover, differences in the content of PO_3_ ions were revealed. The results of ToF-SIMS study correlate with the data obtained by multiphoton microscopy and FLIM, confirming the revealed heterogeneity of hepatocytes in terms of general metabolic activity and intensity of intercellular metabolic processes.

**Conclusion:**

The latest methods of fluorescence bioimaging and mass spectrometry proved to be effective in revealing hepatocyte heterogeneity in terms of metabolic status. The presence of heterogeneity should be taken into account in studying the liver tissue under various conditions with the application of fluorescence bioimaging methods.

## Introduction

The liver is a functional organ performing a wide variety of functions to maintain physiological homeostasis. It dynamically controls the content of metabolites in the body, accumulating nutrients from food [1], in particular, it plays an important role in maintaining constant blood glucose levels. Moreover, the liver carries out complex detoxification processes, it is also involved in bile acid synthesis and excretion, and the synthesis of certain hormones, including insulin-like growth factors. The liver also synthesizes most of the proteins circulating in the blood, including albumin, complement system proteins, and blood coagulation factors [2].

It is known that hepatocytes are spatially heterogeneous and zoned along the portal and central axes of a liver lobule to perform a large number of functions efficiently. This heterogeneity is attributed to the fact that most liver tasks are performed by different subgroups of hepatocytes. Therefore, it is important to take into account in both basic and applied clinical research that cell populations in the parenchyma are heterogeneous in their structure and functions [2–4].

Standard methods for analyzing the character of distribution and heterogeneity of various cell populations (immunohistochemical and morphological analysis) are quite informative, but they do not permit *in vivo* studies on living tissues. Polymerase chain reaction is applied to assess the heterogeneity of tissue cell population by the level of expression of various genes. However, this method is associated with the destruction of cellular structure, which also does not permit studies *in vivo* or on fresh tissue samples. Isotope labeling is also an informative method for detecting various metabolic pathways in heterogeneous cell populations, but its significant drawback is the necessity to add a radioactive label [5].

At present, multiphoton microscopy in combination with fluorescence lifetime imaging (FLIM) is actively employed in biomedical research, including *in vivo* evaluation of metabolic status of various cell types. The method of two-photon excitation fluorescence in the near-infrared range makes it possible to carry out *in vivo* and *ex vivo* studies with a high penetration depth of laser excitation and minimum tissue photodamage [6]. The FLIM method is based on recording the fluorescence lifetime of fluorophores and makes it possible to obtain information not only about the structural characteristics but also about various physicochemical processes in cells [7–10], including hepatocytes [11].

Today, the latest approach for *in vivo* noncontrast assay of metabolic status of various cells using multiphoton microscopy is evaluation of autofluorescence intensity and fluorescence lifetime contributions of various forms of NAD(P)H (nicotinamide adenine dinucleotide) and FAD (flavin adenine dinucleotide) cofactors.

NADH is known to be involved in reactions of the tricarboxylic acid cycle and oxidative phosphorylation. The reduced phosphorylated form (NADPH) is a cofactor in the reactions of biosynthesis of fatty acids and steroids and has antioxidant activity [11]. Depending on whether NADH is in a free or protein-bound state, the cofactor has a short or long fluorescence lifetime [12]. FAD is a part of enzymes involved in electron transport, DNA repair, nucleotide biosynthesis, beta-oxidation of fatty acids, and amino acid catabolism, as well as those involved in the synthesis of other cofactors such as coenzyme A (CoA), coenzyme Q (CoQ). Besides, reduced FADH2 is a part of Complex II of the mitochondrial electron transport chain, taking part in oxidative phosphorylation reactions [13, 14]. A shift in metabolic status (a change in glycolysis or oxidative phosphorylation activity) is known to be accompanied by changes in the contributions of free and bound forms of NADH fluorescence lifetime. Thus, FLIM provides the possibility to assess indirectly the intensity of glycolysis, oxidative phosphorylation, and biosynthetic processes in various functional states of cells [15–18]. Multiphoton microscopy effectiveness for investigating hepatocytes has been confirmed by a number of researchers who analyzed the details of changes in their metabolic status in various liver pathologies, particularly during *in vivo* investigations [19–21]. However, so far these methods have not been applied to study heterogeneity of normal liver hepatocytes in terms of metabolic status.

Time-of-flight secondary ion mass spectrometry (TоF-SIMS) is another promising tissue investigation method providing information on the distribution of lipid and amino acid composition of tissues at the cellular level [22, 23]. ToF-SIMS makes it possible not only to analyze cell components based on their specific chemical structure but also to assess the distribution of cell metabolites of interest based on chemical mapping data [24]. TоF-SIMS has been successfully applied in combination with other methods for liver tissue analysis, particularly in cirrhosis [24] and fatty liver disease [25]. Previously [26], we demonstrated the use of the TоF-SIMS method to detect changes in the ratio of saturated, unsaturated, and polyunsaturated fatty acids in acute cholestasis and chronic liver fibrosis. The main advantage of ToF-SIMS is the absence of necessity for additional staining of tissue samples or addition of various markers, as well as the possibility of targeted analysis of the region of interest.

The FLIM and TоF-SIMS methods have already shown their efficacy for evaluating the degree of cell population heterogeneity, in particular, for determining cellular composition heterogeneity in various types of tumors [27–31].

This paper is devoted to analysis of the degree of hepatocyte heterogeneity in terms of metabolic status, carried out using the latest imaging methods in combination with secondary ion mass spectrometry.

**The aim of the investigation** was to study the possibility of revealing the heterogeneity of normal liver hepatocytes in terms of metabolic status using multiphoton microscopy and mass spectrometry with chemical mapping.

## Materials and Methods

### Laboratory animals

The study was carried out on 25 male Wistar rats weighing 300–400 g. The animals were kept in accordance with the Guide for the Care and Use of Laboratory Animals and the International Principles for Biomedical Research Involving Animals. Experiments with the animals were performed in accordance with the ethical principles of the European Convention for the Protection of Vertebrate Animals used for Experimental and Other Scientific Purposes (Strasbourg, 2006) and approved by the Ethics Committee of Privolzhsky Research Medical University (Nizhny Novgorod, Russia). Before obtaining the liver material, the animal was weighed and then anesthetized with a Zoletil solution at a concentration of 80 mg/kg of body weight. Next, the animal’s liver was completely removed for examination. The obtained material was weighed and washed with saline. After that, samples were prepared for investigation using multiphoton microscopy, morphological analysis, and time-of-flight mass spectroscopy.

### Morphological analysis

To perform histological studies, the liver was fixed in a 10% solution of neutral formalin, dehydrated in alcohols of ascending concentration using the standard technique. Dewaxed sections (5 μm thick) were stained with hematoxylin and eosin according to the standard technique [32, 33]. A Leica DM2500 microscope (Leica Microsystems, Germany) was used to obtain 10 micrographs for each sample without overlapping over the entire surface of the tissue section at 10× and 40× magnification.

### Multiphoton microscopy

*Ex vivo* examination of liver samples obtained within 10 min after organ harvesting was carried out using a laser scanning confocal microscope LSM 880 (Carl Zeiss, Germany) equipped with a Ti:Sa femtosecond laser (repetition rate 80 MHz, pulse duration 140 fs) and time-correlated photon counting system Simple-Tau 152 (Becker & Hickl GmbH, Germany). The average power was about 6 mW. The liver tissue was examined to acquire images of the fluorescence intensity of NAD(P)H and FAD, as well as FLIM images (10 for each animal). For this purpose, a C-Apochromat 40×/1.2 oil immersion objective was used. NAD(P)H autofluorescence was excited at 750 nm, emission was detected in the 450– 500 nm range, FAD autofluorescence was excited at 900 nm, and emission was detected in the 500–550 nm range. Autofluorescence intensity values of cofactors NAD(P)H and FAD were estimated using ImageJ software (integrated density parameter). Accumulation of photons by the FLIM system was carried out for 90 s.

The obtained fluorescence lifetime values of various forms of NAD(P)H were quantified using SPCImage program (Becker & Hickle GmbH). The data were analyzed in the area of cell cytoplasm, excluding the nucleus. The following parameters were assessed for each image: t1 (ps) — fluorescence lifetime of the free form of NADH; a1 (%) — the contribution of the free form of NADH; a2 (%) — the contribution of the bound form of NADH; a3 (%) — contribution of NADPH; a1 + a2 + a3 = 100%.

To analyze the autofluorescence intensity of cofactors NAD(P)H and FAD and the metabolic status of hepatocytes by the FLIM method, 10 fluorescent images were obtained, 30 regions of interest (corresponding to an individual hepatocyte) isolated in hepatocyte cytoplasm in each image.

### Estimating the degree of heterogeneity

To estimate the degree of hepatocyte heterogeneity in terms of metabolic status, the obtained values of a1 and a3 were divided into 15 subgroups:

subgroup 1 — a1 in the range of 50–60% and a3 in the range of 4–6%;

subgroup 2 — a1 in the range of 50–60% and a3 in the range of 6–8%;

subgroup 3 — a1 in the range of 50–60% and a3 in the range of 8–10%;

subgroup 4 — a1 in the range of 50–60% and a3 in the range of 10–12%;

subgroup 5 — a1 in the range of 50–60% and a3 in the range of 12–14%;

subgroup 6 — a1 in the range of 60–70% and a3 in the range of 4–6%;

subgroup 7 — a1 in the range of 60–70% and a3 in the range of 6–8%;

subgroup 8 — a1 in the range of 60–70% and a3 in the range of 8–10%;

subgroup 9 — a1 in the range of 60–70% and a3 in the range of 10–12%;

subgroup 10 — a1 in the range of 60–70% and a3 in the range of 12–14%;

subgroup 11 — a1 in the range of 70–80% and a3 in the range of 4–6%;

subgroup 12 — a1 in the range of 70–80% and a3 in the range of 6–8%;

subgroup 13 — a1 in the range of 70–80% and a3 in the range of 8–10%;

subgroup 14 — a1 in the range of 70–80% and a3 in the range of 10–12%;

subgroup 15 — a1 in the range of 70–80% and a3 in the range of 12–14%.

For each subgroup, the cells were counted, for which the values of a1 and a3 corresponded to a certain range. We built two-dimensional histograms showing the distribution of NAD(P)H and FAD autofluorescence intensity in hepatocytes, and three-dimensional histograms showing the distribution of a1, a2, and a3 values in hepatocytes in the RStudio development environment using the R 4.0.2 language and the car Package library (scatter3d function).

### ToF-SIMS analysis

Analysis of lipid and amino acid compositions of hepatocytes was performed on cryosections of the liver by secondary ion mass spectrometry on a TOF.SIMS 5 device (IONTOF GmbH, Germany). Primary Bi_3_^+^ ions with an energy of 30 keV were used as a source. The analysis was carried out in three modes. In all modes, a low-energy electron gun was activated to avoid sample charging, and the primary ion dose density was maintained below the static SIMS limit (10^13^ ions/cm^2^).

Statistical analysis was performed in the spectroscopic mode. Mass spectra were obtained from a region of 300×300 μm (with a spatial resolution of 5 μm) in both positive and negative ion modes. For each section, 18 measurements were taken from randomly selected non-overlapping regions. Secondary-ion yields were calculated as the intensity of the corresponding peak ratio of lipid to amino acid, normalized to the total number of ions, using the SurfaceLab 6 software (IONTOF GmbH, Germany). Chemical mapping was performed for lipid and amino acid ions identified in the mass spectra in two modes with a spatial resolution of 800 nm and 200 nm, respectively.

### Statistical data processing

Based on the data obtained by the ToF-SIMS method, the mean secondary ion yield and standard deviation were calculated. The distribution was normalized using the graphical method. To identify statistically significant differences, t-test was used. The p<0.05 and p<0.001 values were considered as critical levels of significance. Normality of sample distribution was confirmed using the quantile-quantile plot method. Statistical analysis of the data was performed using Excel and Statistica 10.0 software.

## Results

### Morphological analysis

**Figure 1:**
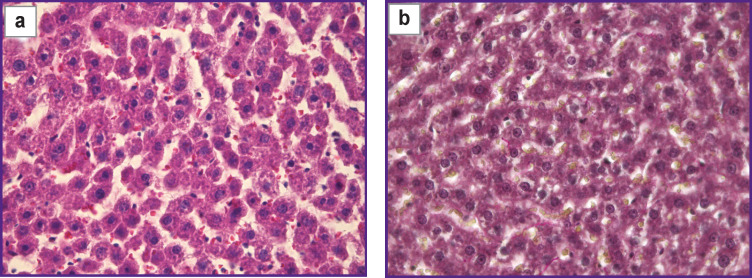
Microimages of histological sections of normal liver tissue: (a) staining with hematoxylin and eosin; (b) staining by the Van Gieson method; 40×

Normal liver tissue sections stained with hematoxylin and eosin, as well as by the Van Gieson method, are shown in Figure 1. Using standard methods of histological analysis, we revealed no heterogeneity in the structural parameters of hepatocytes. The cells were uniformly stained, no signs of edema or degenerative changes were observed.

### Multiphoton microscopy and evaluating the degree of heterogeneity in hepatocyte population of normal liver tissue ex vivo samples

Analysis of the degree of hepatocyte heterogeneity was performed by the FLIM method without reference to particular zones of the liver lobule. This approach was used to prove the presence of heterogeneity in hepatocytes even in random fields of the microscope. Besides, this allowed reducing the analysis time, since the procedure for accurately determining the liver lobule zones is time-consuming and difficult to implement in a clinical setting.

**Figure 2:**
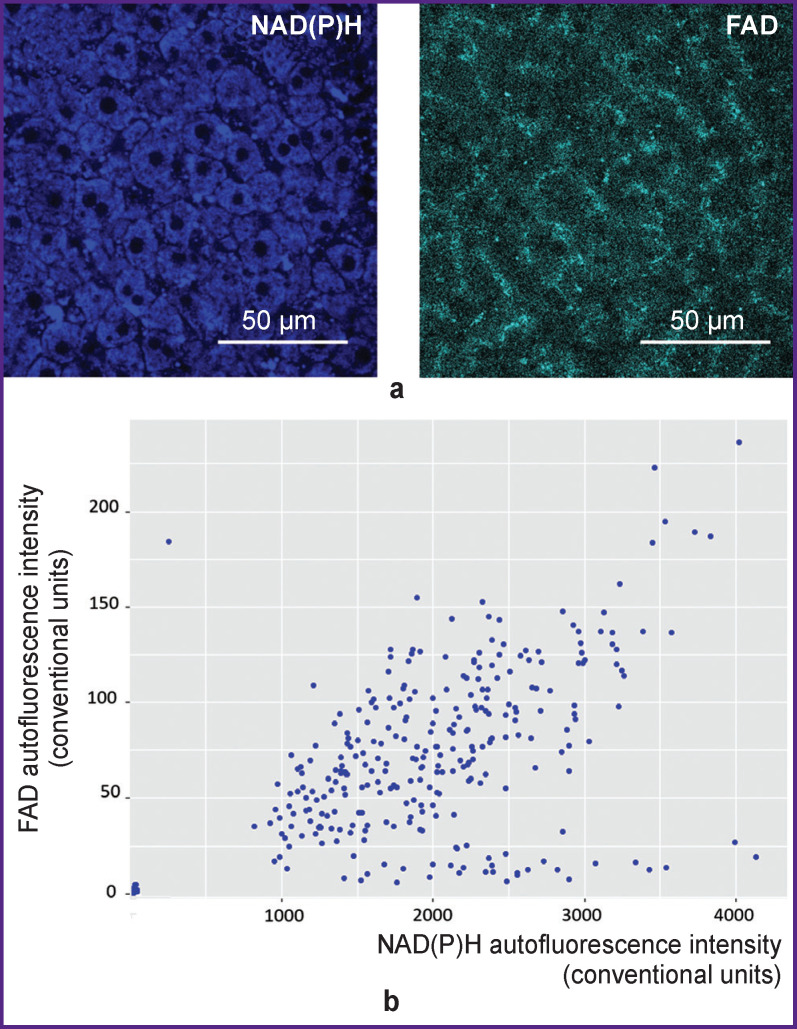
Multiphoton microscopy of normal liver tissue: (a) images of NAD(P)H and FAD autofluorescence in hepatocytes; (b) diagram showing the scatter of autofluorescence intensity of cofactors in the normal liver of linear animals. NAD(P)H: excitation — 750 nm, emission — 455–500 nm; FAD: excitation — 900 nm, emission — 500– 550 nm; field of view — 213×213 μm (1024×1024 pixels)

Thus, to assess the degree of hepatocyte heterogeneity in terms of metabolic status, we obtained data on the autofluorescence intensity of cofactors NAD(P)H and FAD in hepatocytes. An example of liver parenchyma visualization with the use of multiphoton microscopy and distribution of fluorescence intensity values of the cofactors for all studied cells are shown in Figure 2.

The values of autofluorescence intensity of intracellular cofactors suggested a high degree of hepatocyte heterogeneity. Cells with a low-intensity signal of NAD(P)H and FAD autofluorescence represent a population with low total metabolic activity. Cells with a high-intensity autofluorescence signal represent a population with high overall metabolic activity. Figure 2 shows that normal tissue cells of linear animals are heterogeneous in terms of autofluorescence intensity values. Cells with both high and low metabolic activity are present.

To perform a targeted analysis of the metabolic status of normal liver hepatocytes and determine the presence of cell heterogeneity, the values of contributions of free and bound NADH (a1 and a2, respectively), and the contribution of bound NAD(P)H (a3) were obtained. In accordance with the obtained values, 15 subgroups were distinguished, differing in the intensity of various metabolic pathways in cells (glycolysis, oxidative phosphorylation, pentose phosphate pathway, lipogenesis, nucleotide synthesis). Based on the data array obtained by the FLIM method, we constructed a table showing the percentage of hepatocytes in the corresponding subgroup of the total number of cells studied. Selection of hepatocyte subgroups differing in the characteristic values of the contributions of various NAD(P)H forms was based on previous studies devoted to the metabolic status of hepatocytes in the liver in different conditions [26]. The results of the analysis of hepatocyte heterogeneity in terms of metabolic status are presented in the Table.

**Table:** Percentage of various subgroups of hepatocytes in the normal liver of linear animals

a3 (%)	а1 (%)		
	50–60	60–70	70–80
4–5	0.07	0.07	5.27
6–7	0.67	16.13	27.40
8–9	2.40	35.60	2.07
10–11	1.20	6.33	0.47
12–13	0.33	1.53	0.53

It was found that the hepatocyte population of linear animals had high-degree heterogeneity in terms of metabolic status. It was possible to distinguish subgroups of hepatocytes predominating in the sample parenchyma. Subgroups 7, 9, 10, and 13 appeared to be the most significant in terms of quantity. Despite the predominance of the hepatocyte subgroup with a1 values in the range of 60–70% (balance of glycolysis and oxidative phosphorylation processes) in the parenchyma, this range was dominated by cells with increased intensity of biosynthetic processes (a3 values in the range of 8–9%). Moreover, there was also a significant number of hepatocytes with a1 values in the range of 70–80% (a shift in metabolic status towards increased intensity of glycolysis processes), cells with biosynthetic processes of lower intensity prevailing in this range (a3 values in the range 6–7%). The contribution of the hepatocyte population with a1 values in the range of 50–60% was insignificant (a shift in metabolic status towards increased intensity of oxidative phosphorylation processes).

**Figure 3:**
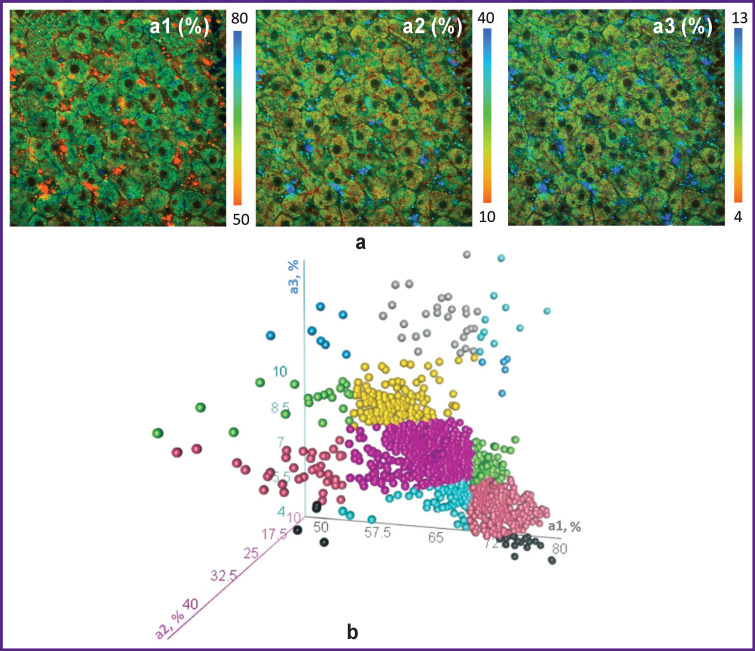
Distribution of contributions of the fluorescence lifetime in normal hepatocytes: (a) FLIM images of various forms of NAD(P)H; field of view — 213×213 μm (512×512 pixels); (b) histogram showing the distribution of contributions from various forms of NAD(P)H. Each color on the scatter diagram corresponds to an individual subgroup of hepatocytes, one point corresponds to one hepatocyte studied

The histogram presenting three-dimensional distribution of contribution values for various forms of NAD(P)H (Figure 3) shows that the hepatocyte population in the normal liver of linear animals is heterogeneous in terms of the three FLIM parameters investigated.

### ToF-SIMS analysis

**Figure 4:**
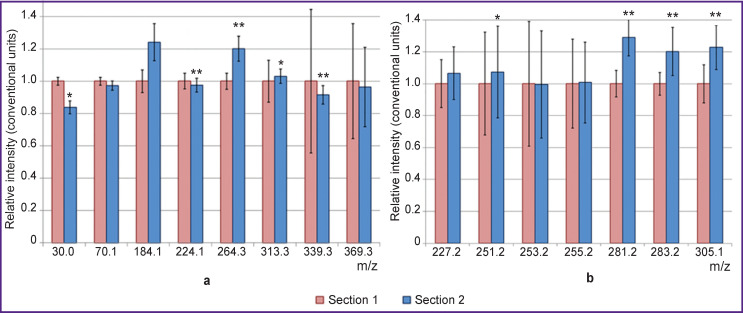
Secondary-ion yields of lipids and amino acids for two sections of the liver: (a) positive ions; (b) negative ions. Data are presented as M±σ, where M is the arithmetic mean, σ is the standard deviation. Statistically significant differences when comparing sections: * p<0.05; ** p<0.001

When performing statistical analysis of secondary ion signals from different liver sections of the same animal (Figure 4), the greatest differences were observed for sphingomyelin ions (the ratio of the ion mass to its charge (m/z) was 104, 184, 264) and the amino acid glycine (m/z — 30).

It is necessary to underline that the coefficients of secondary ion variation differ greatly depending on the ion. For example, standard deviations are minimal within one section for amino acid ions (m/z — 30, 70, 84), while being very significant for cholesterol (m/z — 369), fatty acids (all ions in Figure 4 (b)), monoacylglycerides (m/z — 313, 339), which points to heterogeneity of distribution of these lipids in the same section. It should be noted that monoacylglycerides are most likely fragments of fats, while fatty acids can be fragment ions of both fats and phospholipids.

**Figure 5:**
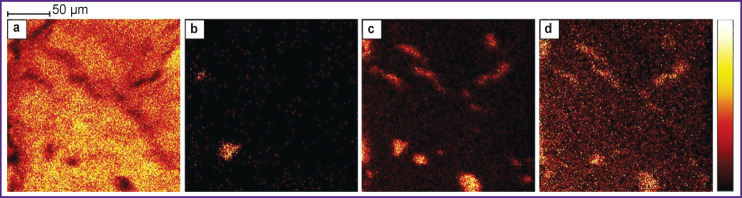
Chemical mapping of lipid distribution in liver sections with 800 nm resolution: (a) phosphatidylcholine and sphingomyelin head group (m/z — 184); (b) cholesterol (m/z — 369); (c) palmitic acid C16:0 (m/z — 255); (d) stearic acid C18:0 (m/z — 283)

**Figure 6:**
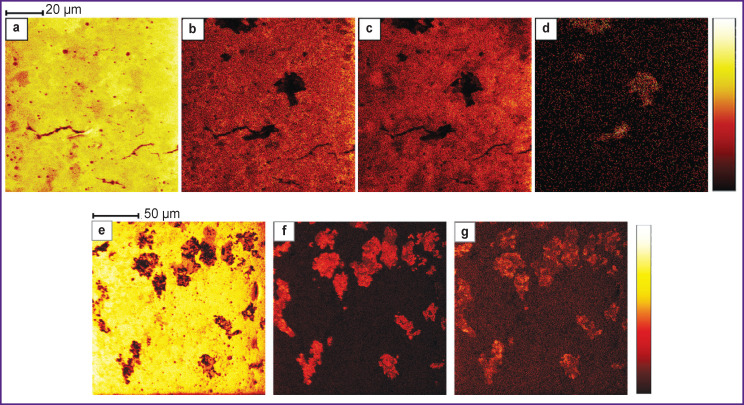
Chemical mapping of lipid distribution in liver sections with 200 nm resolution: positive ions: (a) potassium (m/z — 39); (b) a fragment of proline and glutamic acid (m/z — 70); (c) phosphatidylcholine and sphingomyelin head group (m/z — 184); (d) cholesterol (m/z —369); negative ions: (e) phosphate group (PO_3_) (m/z — 79); (f) palmitic acid C16:0 (m/z — 255); (g) stearic acid C18:0 (m/z — 283)

To assess visually the uneven distribution of chemical composition in hepatocytes at the cellular level, images of sections with chemical mapping at 800 nm (Figure 5) and 200 nm (Figure 6) resolutions were acquired. Distribution of some lipids is shown. To correlate the data, measurements were performed in one region for both positive and negative ions. Some lipids such as phosphatidylcholine and sphingomyelin in Figure 5 are distributed rather evenly, but there are dark spots indicating a drop in signal intensity in this area. Cholesterol and fatty acids, on the other hand, show significant clustering, which provides a signal much higher than the background. These clusters have different shapes, the size varying in the range of 5–70 μm. Among fatty acids, clustering is most significant for acids with 16 carbon atoms (palmitic, palmitoleic acids), but this effect is less significant for fatty acids with 18 carbon atoms (stearic, oleic, linoleic, and linolenic acids). Often cluster locations and shapes are the same, but there are differences as well. Besides, the dark areas in sphingomyelin and phosphatidylcholine images coincide with the light clusters of cholesterol and fatty acids. Since no cellular structure is observed at this resolution, it is difficult to establish what particular clusters belong to. However, these maps explain well the high variation coefficients observed for the intensities of some lipids in the spectroscopic mode.

The 200 nm resolution (see Figure 6) is close to the maximum spatial resolution available for ToF-SIMS. However, in addition to the obvious improvement in image quality, there are also some difficulties when using it. Mass resolution becomes worse and pixel intensity is also degraded. Therefore, to achieve the same intensity values, it is necessary to expose the sample to the primary ions for 16-fold longer time than with the 800 nm resolution. This results in a dose of primary ion irradiation of ~5·10^12^ ions/cm^2^, which is close to the static limit of SIMS. Such high doses, as a rule, lead to degradation of organic and biological material; therefore, analysis in the modes of positive and negative ion detection was carried out for different zones.

Figure 6 (a) shows the distribution of potassium, visualizing the outlines of hepatocytes. Ion distributions in Figure 6 (b) and 6 (c) are similar, although they belong to completely different classes of compounds — amino acids and phospholipids. Dark zones corresponding to a low signal from proline and glutamic acid fragments (see Figure 6 (b)), as well as a low signal from the head groups of phosphatidylcholine and sphingomyelin (see Figure 6 (c)), coincide with the bright zones corresponding to cholesterol accumulations (Figure 6 (d)).

Figure 6 (e) shows the distribution of the PO_3_ phosphate group. This group can be a fragmentary ion of ATP, NAD(P)H, RNA, phosphorylated proteins, phospholipids. The dark zones corresponding to a low signal from phosphate ions match the light zones corresponding to a high signal from fatty acids (Figure 6 (f), (g)), which indicates that the zones with a low signal of the phosphate group PO_3_ have a high signal of palmitic and stearic acid.

Thus, the study revealed the heterogeneity of distribution of various lipids (in particular, cholesterol, fatty acids, and monoacylglycerides) and individual amino acids (in particular, glycine) in the liver tissue. The results obtained may suggest the presence of heterogeneity in the intensity of lipogenesis and amino acid synthesis in hepatocytes of the normal liver.

It is important to note that standard histological methods did not allow revealing the heterogeneity of the hepatocyte population. The use of modern methods of fluorescence bioimaging and mass spectrometry with chemical mapping made it possible to reveal the heterogeneity of the hepatocyte population in terms of metabolic status and distribution of cellular lipids and amino acid fragments.

The results obtained in this work will be used for further developing the criteria for evaluation of the state of liver tissue in pathology based on the analysis of hepatocyte heterogeneity in random fields of view using modern methods of intravital visualization and mass spectrometry with chemical mapping.

## Discussion

From the metabolic point of view, the functional unit of the liver is the hepatic lobule. Blood enters the periphery of the lobule at the portal units and moves radially inward to the draining central vein through sinusoidal blood vessels. As a result, concentration gradients of substances (growth factors, metabolites, and oxygen) are formed, which are dissolved in the blood or secreted by the overlying periportal hepatocytes [34]. The gradient is determined by changes in the composition of blood plasma and oxygenation differences in the bloodstream of the area between the periportal and perivenous zones [35]. The phenomenon of hepatocyte heterogeneity has been studied in many works [34–38]. It was shown that hepatocytes located in the periportal zone (zone 1) specialize in gluconeogenesis and β-oxidation, while glycolysis, lipogenesis, and detoxification processes of high intensity were detected in hepatocytes located around the central vein (zone 3). Consequently, hepatocytes are functionally heterogeneous. Besides, differences in the expression of various genes were found to depend on the location of hepatocytes in the hepatic lobule along the porto-central axis [39]. Interdependent metabolic pathways (e.g., lipogenesis and glycolysis) are colocalized to provide synergistic effects, while opposing pathways are located in different zones, thereby avoiding mutual interference. In general, the heterogeneity of hepatocyte distribution makes it possible to simultaneously perform different and even opposing metabolic functions, providing a flexible adaptation to various conditions [2, 40].

At present, there are data on changes in hepatocyte heterogeneity levels and disruption of zonal arrangement of hepatocytes in various liver pathologies. In particular, the degree of hepatocyte heterogeneity in terms of metabolic status changes with the development of alcoholic fatty liver disease, which is associated with impaired metabolism of fatty acids in hepatocytes [41]. There is evidence of selective deposition of triglycerides (TGs) in pericentral hepatocytes (pericentral steatosis) and increased lipogenic activity in pericentral areas. In addition, the intensity of β-oxidation was found to increase in pericentral hepatocytes with the development of pathology, while in control animals the intensity of this process was higher in the periportal zones of the liver lobe [41].

Development of nonalcoholic fatty liver disease is accompanied by imbalance in the metabolism of fatty acids [42], which leads to inhomogeneous TG deposition in the liver. Besides, the distribution of enzymes involved in the metabolism of phosphatidylcholine and gluconeogenesis reactions was reported to change in this case [43].

Development of steatohepatitis was found to be accompanied by a zonal increase in lipid peroxidation and oxidative stress in combination with reduced oxygen tension on mitochondrial membranes. This change is associated with the development of mitochondrial dysfunction in individual hepatocytes [44].

Progression of chronic liver diseases leads to the development of cirrhosis, when zonal distribution of hepatocytes is completely lost and glutathione synthetase synthesis practically stops (disruption of the glutathione cycle) [45].

Thus, evaluation of the degree of hepatocyte heterogeneity with modern imaging methods becomes more important when conducting an intraoperative examination of the state of liver tissue, in particular, when identifying background diseases.

Currently, fluorescence bioimaging methods are widely used to assess the metabolic status of cells, in particular, tumor and stem cells [45–50]. Besides, there has been reported effectiveness of multiphoton microscopy methods in combination with FLIM for the analysis of the metabolic status of hepatocytes in normal conditions and various liver pathologies [19, 27].

To reveal hepatocyte heterogeneity in terms of metabolic status, this study involved finding the fluorescence intensity values of cofactors NAD(P)H and FAD, the contributions of free and bound forms of NADH (a1 and a2), as well as the contribution of the bound form of NAD(P)H (a3) from various parts of liver tissue. We did not conduct targeted assessment of liver lobule zones, because performing a targeted analysis is a complex, time-consuming process increasing the duration of the procedure. Therefore, “blind” imaging is a more feasible approach in clinical practice. Yet, even without regard to the targeted analysis of zones, we revealed a high degree of hepatocyte population heterogeneity as to the intensity of intracellular processes of oxidative phosphorylation and glycolysis, and the intensity of biosynthetic processes in random fields.

ToF-SIMS provides the opportunity to study lipid and amino acid composition of liver tissue. In this work, we performed a statistical analysis of the lipid and amino acid composition of hepatocytes in random fields. A significant variation in intensity was found for some ions of fatty acids, monoacylglycerides, cholesterol, depending on the location of analysis. Chemical mapping has provided the possibility to confirm this result. Fatty acids, cholesterol are concentrated in individual cells. These cells are also characterized by reduced intensity of phosphatidylcholine and amino acids (proteins). Moreover, cells with an increased lipid signal also differ: some hepatocytes have an increased signal of single fatty acids only, others — of a certain combination of fatty acids, while some hepatocytes have also an increased signal of cholesterol. Triacylglycerides are most likely the source of increased fatty acid signal. By contrast, the level of phospholipids whose content correlates with the intensity of the phosphate ion is reduced in these cells. Interestingly, short-chain fatty acids (16 carbon atoms) exhibit a more significant contrast on chemical maps compared to long-chain fatty acids (18 and 20 carbon atoms). This indicates that short-chain fatty acids tend to accumulate more strongly in the individual hepatocytes. No distribution tendencies were found when making comparisons in terms of fatty acid unsaturation degree. The results obtained are in line with the literature data describing the presence of heterogeneity in terms of biosynthetic process intensity in hepatocytes: in particular, there was revealed increased lipogenesis in separately located hepatocytes [51]. It was shown that the intensity of lipid metabolism was uneven in liver cells [52]: in particular, the rate of fatty acid synthesis was found to be higher in pericentral hepatocytes, while the rate of β-oxidation was higher in periportal liver cells. Katz et al. [53] revealed higher activity of the lipogenic enzymes acetyl-CoA carboxylase and ATP citrate lyase in the pericentral areas, which was shown in a rat model. Besides, the activity of carnitine palmitoyltransferase 1, the key β-oxidation enzyme, was found to be higher in periportal hepatocytes. Carnitine palmitoyltransferase 1 also demonstrates lower sensitivity to inhibition by lipogenic intermediate malonyl-CoA in the periportal zones compared to the pericentral zones [54]. Esterification of fatty acids and synthesis of very-low-density lipoproteins are known to be somewhat higher in hepatocytes localized in the pericentral zones [41, 55]. It has been suggested that the uneven distribution prevents TG accumulation, thereby avoiding the negative consequences of increased lipogenesis in this population of hepatocytes. However, the same study found no differences in the rate of TG excretion for hepatocyte fractions from the periportal and pericentral zones of the liver.

The obtained results of the ToF-SIMS analysis are consistent with the FLIM data demonstrating increased contribution of the bound NADPH form in individual cells, which is associated with the increase in the intensity of biosynthetic processes (mainly lipogenesis) in cells.

Besides, the TоF-SIMS method used in our work made it possible to establish uneven distribution of PO_3_ ions in hepatocytes. The PO_3_ ion is known to be a part of ATP and NAD(P)H. In this regard, different concentrations of this ion in individual hepatocytes can also be associated with different intensities of cellular metabolic activity. This result is consistent with the data of metabolic imaging with multiphoton microscopy that has been applied to reveal hepatocyte heterogeneity in terms of metabolic activity and intensity of oxidative phosphorylation and glycolysis processes.

Thus, fluorescence bioimaging and mass spectrometry methods seem to be promising for analyzing the structural and functional state of liver tissue, and for revealing the heterogeneity of hepatocyte populations in liver tissue *in vivo*. It has been proven that the hepatocyte population is heterogeneous even when analyzed in random fields of view that correspond to no specific zone of the hepatic lobule. It is necessary to take into account the phenomenon of heterogeneity when analyzing the metabolic status of liver tissue cells. There are plans for future development of an algorithm for analyzing the heterogeneity of metabolic and chemical composition of liver tissue based on a random set of hepatocytes.

## Conclusion

Multiphoton microscopy allows performing “blind” visualization to reveal hepatocyte heterogeneity in terms of metabolic status under normal conditions based on the autofluorescence intensity of intracellular cofactors NAD(P)H and FAD, as well as data on the fluorescence lifetime and contributions of various forms of NAD(P)H. The TоF-SIMS method shows the presence of hepatocyte heterogeneity in terms of amino acid and lipid composition, which points to various intensities of synthetic processes in the individual hepatocytes. It can be used to identify differences in the content of PO_3_ ions. The results of the ToF-SIMS analysis are consistent with multiphoton microscopy data and confirm hepatocyte heterogeneity in terms of metabolic activity and intensity of oxidative phosphorylation, glycolysis, and synthetic processes.

The latest methods of fluorescence bioimaging and mass spectrometry proposed in this study are effective in revealing hepatocyte heterogeneity in terms of metabolic status and provide the possibility to estimate the presence of heterogeneity during examination of liver tissue under various conditions, and could be applied in clinical practice.
